# Cystic Echinococcosis of the Bone in Kazakhstan

**DOI:** 10.1155/2018/9682508

**Published:** 2018-09-18

**Authors:** Tommaso Manciulli, Aigerim Mustapayeva, Konrad Juszkiewicz, Ekaterina Sokolenko, Zhaksylik Maulenov, Ambra Vola, Mara Mariconti, Gani Serikbaev, Amangul Duisenova, Enrico Brunetti, Zhamilya Zholdybay

**Affiliations:** ^1^Ph.D. in Experimental Medicine, University of Pavia, Pavia, Italy; ^2^Department of Clinical, Surgical, Diagnostic and Pediatric Sciences, University of Pavia, Pavia, Italy; ^3^Department of Visual Diagnostics, Asfendiyarov Kazakh National Medical University, Almaty, Kazakhstan; ^4^Department of Infectious and Tropical Diseases, Asfendiyarov Kazakh National Medical University, Almaty, Kazakhstan; ^5^Department of Pathology, Cytology and Molecular Pathology of Tumors, Kazakh Institute of Oncology and Radiology, Almaty, Kazakhstan; ^6^Center of Bone Tumors, Soft Tissue and Melanoma, Kazakh National Institute of Oncology, Almaty, Kazakhstan; ^7^Department of Infectious Diseases, IRCCS San Matteo Hospital Foundation, Pavia, Italy

## Abstract

Cystic echinococcosis (CE) is a parasitic zoonosis caused by *E. granulosus* primarily affecting the liver and lungs. CE of the bone is by far the most debilitating form of the disease and is very difficult to manage as it mimics malignant tumors. We reviewed bone CE cases admitted to a reference oncological hospital in Kazakhstan from January 2010 to February 2017. Among eight patients, the mean age was 33.5 years, and the male/female ratio was 1 : 3. Patients were examined by X-ray (8/8), CT (7/8), and MRI (3/8). CE was in the spine (2 cases), pelvis (3 cases), and long bones (humerus, tibia, and femur; one case for each). All patients were treated surgically. No perioperative albendazole was administered. No patient received albendazole afterwards. The mean hospital stay was 25 days. Interventions are urgently needed to assess the burden of CE in Kazakhstan and to inform clinicians of the existence of the disease.

## 1. Background

Cystic *echinococcosis* (CE) is a parasitic disease caused by the cestode *Echinococcosus granulosus.* Its life cycle involves two hosts: dogs (but other carnivores as well) as the definitive host and sheep (and other herbivores) as intermediate hosts [1, 2]. Humans are intermediate incidental hosts or dead-end hosts [1, 3]. Adult parasites are found in the intestine of definitive hosts. The eggs of the parasite are shed with the host's feces into the environment where the intermediate host, usually a sheep (or other herbivores), gets infected when grazing on the contaminated ground. After ingestion of the egg, the embryo (oncosphere) hatches, penetrates the intestinal mucosa, enters into the host's circulatory system (via venous and lymphatic pathways), and develops into the characteristic vesicular metacestode when reaching a suitable anatomical site, assuming the intermediate host's immune system is unable to destroy the oncosphere [1]. This stage of the parasite is typically a unilocular, fluid-filled cystic lesion (“hydatid” and “hydatid cyst”), which grows (increasing in diameter from 1 to 5 cm per year) within the affected organ and harbors the protoscolices [2, 4]. When the definitive host feeds on the infected viscera, the cycle is complete [1]. In humans, the liver is the most frequent location (70% of cases), followed by the lungs (20% of cases), but any organ can be involved [1, 2, 4]. The nervous system is affected in 3% of cases, and the bone in 1 to 4% of cases [5]. The infection may remain asymptomatic for a very long time or manifest as a severe and debilitating condition [3, 4]. To get a sense of the magnitude of the problem, over 250,000 disability-adjusted life years are lost each year because of this zoonosis. CE also has a significant economic impact, with at least $141,605,195 millions of dollars lost annually in animal production [3, 6, 7]. Despite all of the above, CE remains a neglected disease [3, 6, 7]. Osseous CE is one of the most severe forms of the disease [2, 5]. Unlike in other organs, where a cyst with a clear cleavage plan is formed, bone CE spreads with an erosive/infiltrating pattern along the medullary and trabecular channels [8]. The trabeculae are slowly reabsorbed due to pressure without any cortical extension [8]. The cysts then extend to the surrounding soft tissues if the bone cortex is eroded [8]. Vertebral echinococcosis is the most frequent form with 50% of osseous cases [5]. The hip and hip joint follow with 30% of cases, while the remaining 20% is seen in long bones [2]. The paucity of available data does not allow any reliable indications on clinical management [2, 4, 5]. The diagnosis of bone echinococcosis is primarily based on radiological and histopathological findings [5, 8]. However, this diagnosis is most often made postoperatively as the unspecific radiological aspects of the disease can simulate a variety of conditions, from inflammatory to neoplastic processes [5,9–12]. All this renders this disease extremely difficult to manage even for reference centers. In Kazakhstan, CE remains endemic. The country has seen a sharp, 5-fold rise in the incidence of the disease, according to a survey published in 2010, since 1995, from 1.2 to 6.7 per 100,000 people [13], with most cases being seen in rural settings.

## 2. Materials and Methods

This retrospective analysis was carried out at the Kazakh Institute of Oncology and Radiology (KazIOR), Almaty, Kazakhstan. Records of patients diagnosed with CE of the bone between 1 January 2010 and 1 February 2017 were included in the study. Patients with a final diagnosis different from CE were not considered for this study. The KazIOR is an oncology reference center in Kazakhstan. The hospital has 430 beds and manages 8500 patients each year.

## 3. Study Variables

For each patient, we collected demographic data (name, surname, date of birth, region, village, and district of origin), clinical data (symptoms at presentation, preoperative radiological, serological, or pathological data, and preoperative diagnosis), data on the presence or absence of other CE lesions, treatment data (surgery Y/N, albendazole Y/N, secondary CE prophylaxis during surgery Y/N, and use of prosthetics Y/N), treatment outcomes (recurrence Y/N and permanent or transient disabilities), and data on the duration of the hospital stay.

## 4. Results

**Figure 1:**
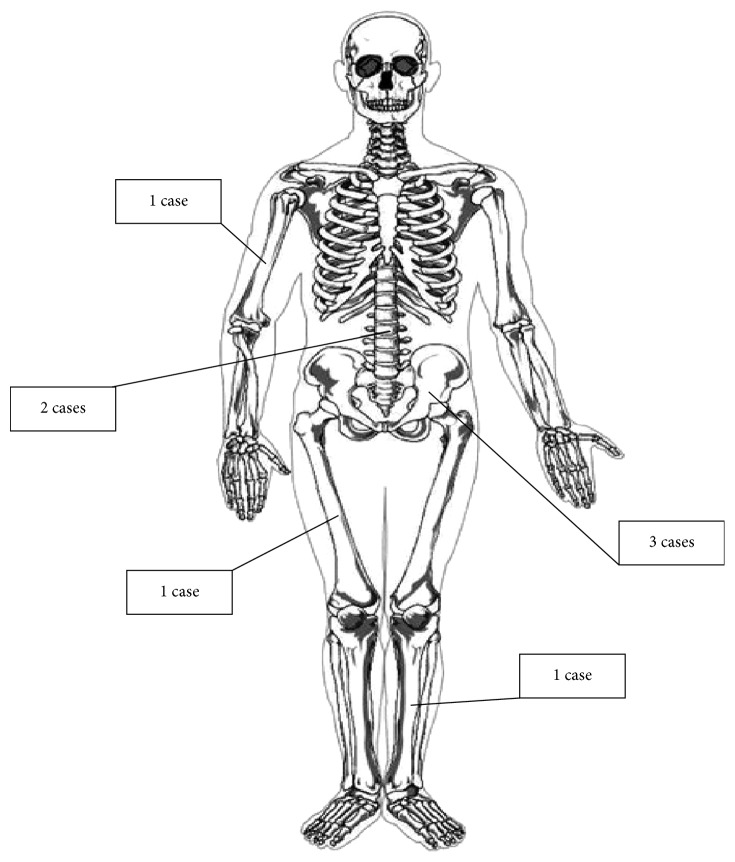
Absolute numbers of the anatomical distribution of bone CE lesions in our study.

Of seventeen patients seen with a diagnosis of CE, eight patients matched our inclusion criteria. CE was present in the spine (two patients), pelvis (three patients), humerus (one patient), femur (one patient), and tibia (one patient)(Figure 1). The median patient age was 33.5 years (range 19–55). Six patients were female and two were male.

Presenting symptoms included pain of the involved segment for all patients, edema (four patients), and difficulty walking (four patients). Seven patients were examined by X-ray, six patients underwent a CT scan, and three patients underwent an MRI. Seven patients underwent an ultrasound examination of the affected segment. In all cases, a malignant tumor of the bone was initially suspected, and all patients were treated with surgery. Despite all patients receiving pathological diagnosis of infection with *E. granulosus*, no patient received albendazole as part of the clinical management. The median hospital stay was 22 days. Only one patient is currently undergoing a regular follow-up. Complications were primarily considered a consequence of surgical treatment, as one patient with CE in the spine developed paraplegia of the legs after the intervention, and another patient sustained a permanent reduction in length of his right leg.

## 5. Discussion

Eight bone CE cases were seen in a single hospital in an endemic region. In our series, the most frequent localization was the pelvis, followed by the spine and long bones. Only one patient presented with a CE cyst in organs other than the bone, consistent with the literature where for 40% of patients bone is the only location [2]. Such a high number of cases of bone CE in a single center confirms that CE is highly endemic in Kazakhstan. While the low number of patients with other localizations of CE seem to contradict this conclusion, it should be noted that all correctly diagnosed CE cases in the Almaty region are treated at a dedicated center. However, knowledge about this disease is lacking as shown by the fact that CE was never included in the differential diagnosis in any of the patients in our cohort. Although CE of the bone is believed to be the consequence of a primary infection, some reported a coexistence of visceral and bone CE locations in 30–45% of cases [14]. In bone CE, an early diagnosis is crucial to improve therapy outcomes and limit the damage caused by this chronic entity [5, 14]. In our series, only one patient underwent a US scan of soft tissues surrounding the affected segment, and only three patients underwent an MRI. Even more worrisome is the fact that no patient received albendazole as part of their clinical management, and only one patient of our cohort is currently undergoing a regular follow-up, a key element of clinical management given the high number of relapses. This is particularly important in bone CE where surgery is a complex, high-risk procedure.

## 6. Conclusions

Our data confirm that bone CE is a highly debilitating disease and a serious clinical challenge as it is frequently misdiagnosed and treated inadequately. In our series, all cases were mistaken for malignant tumors after radiological examinations. Larger studies are needed to infer general conclusions about the pathology and natural history of the disease, as well as to build consensus for the management of this form of CE. Interventions are urgently needed to assess the burden of CE in Kazakhstan and to inform clinicians of the existence of the disease.
